# Regulation of microglia polarization after cerebral ischemia

**DOI:** 10.3389/fncel.2023.1182621

**Published:** 2023-06-08

**Authors:** Hao Wang, Jingjing Li, Han Zhang, Mengyao Wang, Lifang Xiao, Yitong Wang, Qiong Cheng

**Affiliations:** ^1^Key Laboratory of Neuroregeneration of Jiangsu and Ministry of Education, Jiangsu Province Co-innovation Center of Neuroregeneration, NMPA Key Laboratory for Research and Evaluation of Tissue Engineering Technology Products, Nantong University, Nantong, China; ^2^School of Medicine, Nantong University, Nantong, China

**Keywords:** microglia, polarization, neuroinflammation, autophagy, cerebral ischemia

## Abstract

Stroke ranks second as a leading cause of death and permanent disability globally. Microglia, innate immune cells in the brain, respond rapidly to ischemic injury, triggering a robust and persistent neuroinflammatory reaction throughout the disease’s progression. Neuroinflammation plays a critical role in the mechanism of secondary injury in ischemic stroke and is a significant controllable factor. Microglia activation takes on two general phenotypes: the pro-inflammatory M1 type and the anti-inflammatory M2 type, although the reality is more complex. The regulation of microglia phenotype is crucial to controlling the neuroinflammatory response. This review summarized the key molecules and mechanisms of microglia polarization, function, and phenotypic transformation following cerebral ischemia, with a focus on the influence of autophagy on microglia polarization. The goal is to provide a reference for the development of new targets for the treatment for ischemic stroke treatment based on the regulation of microglia polarization.

## 1. Introduction

Based on data from the WHO, stroke is the second most common cause of death and lifelong disability worldwide, with ischemic stroke accounting for 80–90% of all strokes (Fukuta et al., 2017; Henderson et al., 2018; Barthels and Das, 2020). The inflammatory response is an early event in the cascade of cerebral ischemia-reperfusion injury and is present throughout the disease course, including during convalescence (Chamorro et al., 2016). Microglia, which are innate immune cells in the brain, play a central role in the onset and persistence of inflammation (Hu et al., 2012). Selective ablation of proliferating microglia has been shown to worsen cerebral ischemic injury, indicating that the presence of microglia is necessary to mitigate ischemic injury (Lalancette-Hébert et al., 2007). In fact, microglia play dual and opposite roles in the progression of inflammatory responses in ischemic stroke (Huang and Feng, 2013). The activated microglia are classified into two phenotypes: the pro-inflammatory M1 phenotype and the anti-inflammatory M2 phenotype (Xue et al., 2021). However, an increasing number of studies have shown that this binary classification oversimplifies microglia activation, as the two polar phenotypes often overlap and can be transformed by both intracellular and extracellular factors during disease progression. These factors, which regulate the transformation of M1/M2 phenotype, act as molecular switches in signaling pathways and are crucial targets for regulating neuroinflammatory responses to improve prognosis in cerebral ischemia.

Autophagy is a crucial degradation system in eukaryotic cells, whereby autophagosomes enwrap damaged proteins or organelles within their bilayer structure and transport them to lysosomes (animals) or vacuoles (yeast and plants) for degradation (Parzych and Klionsky, 2014). In ischemic stroke, both oxidative stress and inflammation can directly trigger autophagy (Zang et al., 2020; Zhu Y. et al., 2021). Properly regulated autophagy can promote brain injury recovery by reducing oxidative stress and inflammatory responses. Dysfunctional autophagy can exacerbate brain damage, while excessive autophagy can lead to autophagy-dependent cell death (Wang P. et al., 2018; Denton and Kumar, 2019). Autophagy can regulate the polarization and function of microglia, but its mechanism remains controversial. Knocking out Atg5, an autophagy-associated protein, is sufficient to trigger the M1 phenotype without inflammatory stimulation. Autophagy was once thought to act as an endogenous brake on M1 polarization in microglia (Jin et al., 2018). However, recent studies have found that Atg5 in microglia does not play a role in autoimmune neuroinflammation in mice (Srimat Kandadai et al., 2021). Studies in ischemic stroke suggest that microglial autophagy may influence the pathological process of neuroinflammation by regulating polarization.

In this review, we examine the signaling molecules and mechanisms closely associated with the regulation of microglia polarization following cerebral ischemia, with a particular focus on the impact of autophagy. Our goal is to identify potential targets for the development of novel drugs based on the regulation of microglia polarization, which could ultimately improve the prognosis of cerebral ischemia.

## 2. Resting microglia and their normal function

Microglia originate from primitive myeloid progenitor cells, which leave the yolk sac at 8.5–9 days of embryo development and enter the neural tube through the primitive blood flow to the CNS. They acquire genealogy-specific gene expression and differentiate into mature microglia (Ginhoux et al., 2010). In the healthy CNS, microglia account for 5–12% of the total number of cells, with varying proportions in different brain regions (Lawson et al., 1990). Under stable condition, microglia are found in the CNS parenchyma, while CNS-associated macrophages (CAMs) are present in the CNS boundary region, such as the perivascular space, meninges, and choroid plexus. Microglia and CAMs express several common markers, including CD11b CX3CR1 (CX3 chemokine receptor 1), and CD45 (hematopoietic marker). Therefore, they are often grouped together as microglia/macrophages to discuss their activation and function (Hickman et al., 2013). However, recent findings from single-cell sequencing studies have revealed distinct expression profiles of microglia and CAMs. Microglia specifically express P2yr12, Tmem119, Fcrls, Siglech, Slc2a5, SalI1, Hexb, and Trem2, while CAMs specifically express Lyve1, Siglec1, and Mrc1. This highlights the transcriptional and epigenetic differences between microglia and other bone marrow-derived macrophages in the brain (Konishi et al., 2017; Masuda et al., 2020a,b). In this review, we will focus on microglia and their activation and function in cerebral ischemia.

Normal microglia have a highly branched morphology and are commonly referred to as resting microglia. However, recent *in vivo* two-photon microscopy studies have revealed that these so-called resting microglia are not completely stationary but are dynamic and highly responsive to their environment (Yu et al., 2020). The maintenance of microglial quiescence depends in part on neuronal and astrocytic signaling. Resting microglia establish direct contact with neuronal synapses approximately once per hour, enabling them to monitor synaptic function and act as organizers, facilitating synaptic formation and maturation (Nimmerjahn et al., 2005; Kato et al., 2019). Microglia-mediated synaptic pruning and plasticity play an important role in CNS physiology. During early brain development, approximately 20–80% of long axonal neurons undergo apoptosis and are rapidly eliminated by microglia (Sierra et al., 2010). Microglia secrete extracellular signals such as cytokines, hormones, neurotransmitters, or growth factors that bind to specific neuronal receptors to promote neuron survival or initiate programmed cell death, thereby regulating the number of CNS neurons (Aarum et al., 2003; Morgan et al., 2004).

Microglia are not only involved in regulating tissue development, structural refinement, and maintaining the neural environment but also maintain brain homeostasis by removing pathogens, dead cell debris, and abnormal proteins, as well as participating in injury response and subsequent remodeling and repair processes (Orihuela et al., 2016). Microglia use a series of immune receptors, such as NODs, NLRs, TLRs, and SRs, to identify adverse stimuli and withstand self and non-self injuries (Hickman et al., 2018). In monitoring mode, once they encounter a danger signal, microglia rapidly activate and undergo morphological changes, such as enlarging the cell body, shortening the process, and increasing phagocytic activity (Tsukahara et al., 2020). These functions determine the crucial role of microglia in the brain development and various CNS diseases.

## 3. Phenotype and morphological changes of activated microglia

Microglia activation is involved in the pathogenesis of almost all CNS diseases. Furthermore, the response of microglia to stimuli is influenced by age and sex. In C57BL/6 mice, the expression levels of iNOS, TNF-α, and Arg-1 were higher in microglia at 3 days of age, while the levels of CD11b, TLR4, and FcRγ increased in microglia at 21 days of age. The expression levels of pro-inflammatory factors in microglia were low in adult mice but increased with age. Interestingly, at 3 days of age, females expressed more inflammatory factors than males, even though there was no difference in estrogen levels between them at this age (Crain et al., 2013).

Activated microglia can polarize in two rather different directions, depending on the initial stimulus that caused the activation. Exposure to bacteria-derived products (such as LPS) or infection-associated signals (such as IFN-γ) promotes M1-type polarization of microglia, which is known as classic activation (Kalkman and Feuerbach, 2016). M1-type microglia produce pro-inflammatory cytokines such as IL-1α, IL-1β, IL-6, IL-12, and TNF-α, REDOX molecules like NADPH oxidase, phagocytic oxidase, and iNOS, as well as MHC-II, chemokines such as CCL2, CXCL9, CXCL10, and large amounts of ROS, which promote inflammatory responses and play neurotoxic roles (Ransohoff and Brown, 2012; Gyoneva and Ransohoff, 2015).

Interleukin 4 and IL-13 can induce alternative activation of microglia, also known as M2-type polarization, which plays a role in inflammation inhibition, tissue remodeling, angiogenesis, and immune regulation. Unlike classical activation, M2-type polarization leads to a phenotypic change that promotes neuroprotection. M2-type polarization can be further divided into M2a, M2b, and M2c subclasses (Walker and Lue, 2015). M2a microglia produce anti-inflammatory factors such as IL-10 and IGF-1, up-regulate the expression of Arg-1, CD206, and Ym1, inhibit NF-κB, and enhance phagocytosis (Martinez and Gordon, 2014). M2b microglia are induced by the triggering of immunoglobulin FC-γ receptors, TLRs, IL-1R, and immune complexes on the surface of microglia. They down-regulate the expression of IL-12 and increase the secretion of IL-10. The M2b subclass is considered to be a transitional activation state with some characteristics of both M1 and M2 type and is a potential regulator of M2 type (Gensel and Zhang, 2015). M2c polarization can be induced by IL-10 and glucocorticoid, up-regulating the expression of TGF-β, and participating in tissue remodeling and matrix deposition after inflammation (Siddiqui et al., 2016). Table 1 shows the triggers of microglia with different phenotypes and their typical markers.

**TABLE 1:** Triggers and biomarkers for microglial phenotypic diversity.

Triggers	Phenotypes	Markers	References
LPS, INF-γ	M1	iNOS, TNF-α, IL-6, IL-1β, IL-12, IL-23, CCL5, CCL11, CD14, CD16, CD32, CD86	Ransohoff and Brown, 2012; Gyoneva and Ransohoff, 2015
IL-4, IL-13	M2a	Arg-1, CD206, Ym1, PPAR, TREM2	Martinez and Gordon, 2014; Walker and Lue, 2015
FcγR, TLRs, IL1R	M2b	CD32, CD64	Gensel and Zhang, 2015
IL-10, IL-4, glucocorticoid (GC)	M2c	CD163, CD206, CCR2, IL-4Rα, TGF-β	Siddiqui et al., 2016

Microglia exhibit a wide range of phenotypic diversity, and their morphology is closely related to their function. Microscopic observation reveals that resting microglia have small cell bodies and very fine, long ramifications, while activated microglia have enlarged cell bodies and shortened ramifications (Lier et al., 2021). Amoeboid microglia are characterized by swelling of the cell body and complete contraction of the protrusions (Doorn et al., 2014). The highly branched and amoeboid morphologies represent two extremes of the microglia morphological spectrum, with a variety of transitional states existing between them. These states likely reflect disease-specific functional cell states and are closely related to their precise role in the damaged brain (Salamanca et al., 2019).

**FIGURE 1:**
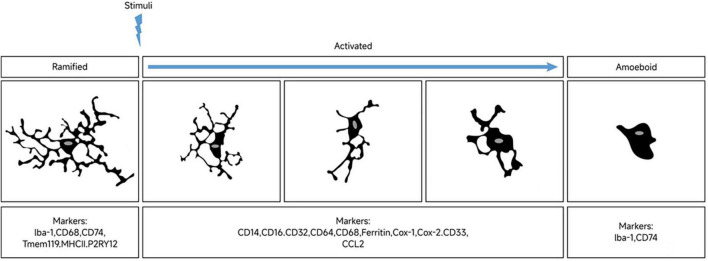
Morphological changes of microglia upon stimulation and markers expression across polarization phases. In their resting state, microglia display a highly branched morphology. Stimulation results in shorter branches and enlarged cell bodies. Microglia exhibit different polarization phenotypes in response to distinct triggering signals. At opposite ends of the morphological spectrum are the highly branched and amoeboid microglial phenotypes, with a diverse range of morphologies observed between them. Markers associated with microglial polarization are expressed differentially across these distinct phenotypic phases.

Since most markers may be expressed simultaneously in different microglial functional states, it is impractical to identify phenotypes with a single marker. For example, while Iba-1 is a classic marker of microglial activation, it cannot distinguish between microglia with different polarizing phenotypes (Ito et al., 1998). Tmem119 is expressed in both resting and dystrophic microglia, while CD68 expression is significantly increased in both activated and amoeboid microglia. Similarly, ferritin is expressed in both activated and dystrophic microglia (Lier et al., 2021). Therefore, when differentiating microglia in different functional states, appropriate markers should be selected and combined with more than two markers to increase the accuracy of identification. Figure 1 displays the morphological changes of resting microglia after stimulation and the markers in different phases.

The classification of microglia function based on morphological characteristics often involves multiple quantitative parameters. However, this approach may be susceptible to biases arising from artificial selection, which can ultimately affect the accuracy of the classification (York et al., 2018; Kyriazis et al., 2019). Leyh et al. (2021) developed a novel method to classify microglia based on morphological assessment using convolutional neural networks. They focused on four microglia morphologies found in the hippocampus and cortex of mice: branched, rod-shaped, activated, and amoeboid microglia. This classification method was validated in a mouse model of ischemic stroke, indicating that machine learning can be employed as an objective and time-efficient approach to characterize microglial changes in healthy and diseased mouse models. Moreover, it may also serve as a valuable tool for analyzing human brain autopsy samples. Salamanca et al. (2019) developed a pipeline called the microglia and immune cells morphologies analyzer and classifier (MIC-MAC), which semi-automatically segments, extracts, and classifies all microglia and immune cells labeled in large 3D confocal image stacks of mouse and human brain samples. MIC-MAC is a precise diagnostic tool capable of automatically characterizing and classifying the 3D morphologies of thousands of individual microglial cells, enabling rapid, unbiased, large-scale analysis of microglia morphological states in mouse models and human brain autopsy samples.

Apart from variations in inflammatory factors and microenvironment, are there discernible morphological differences between the M1 and M2 phenotypes of microglia? Evidence suggests that a mixture of M1-type activation markers (CD74, CD40, CD86, and CCR7) and M2-type activation markers (CCL22 and CD209) can be detected in the brains of individuals with multiple sclerosis (MS), but it remains unclear whether these different activation states correspond to unique morphological features of microglia (Peferoen et al., 2015). This indicates that the expression of both M1-type and M2-type markers is dynamic and that microglia can switch between the M1 and M2 phenotypes. In the brain of patients with AD, the co-expression of both M1-type and M2-type markers, including M2a, M2b, and M2c, can also be observed (Sudduth et al., 2013). Therefore, as activated microglia, it is difficult to distinguish between the M1 and M2 phenotypes based solely on morphology. It is necessary to associate their morphology, markers, and functional states together for accurate classification.

## 4. Polarization dynamics of microglia after ischemic stroke

Microglial activation can be detected clinically in the acute, subacute, and chronic stages of ischemic stroke (Gulyás et al., 2012). In animal models of ischemic stroke, microglial activation varies dynamically over time and space at different stages, and is correlated with the severity of the stroke (Colton, 2009).

**FIGURE 2:**
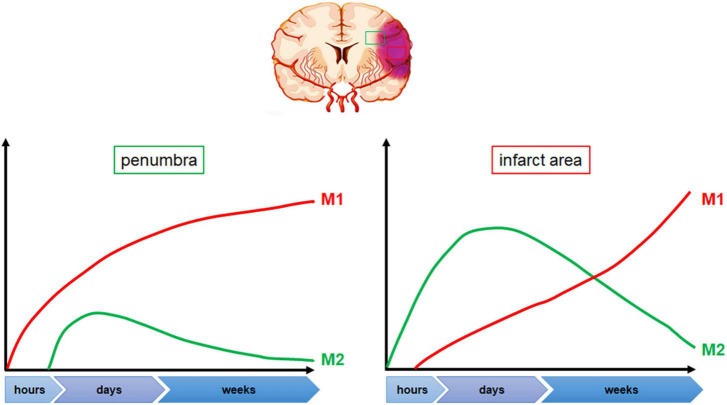
Dynamic changes in microglia polarization following cerebral ischemia. Following stroke onset, microglia polarization shifts toward the M2 phenotype within the first few hours, with predominant accumulation in the ischemic area. Peak M2 polarization is observed between 3 and 5 days post-stroke, following which microglia gradually transition toward the M1 phenotype, primarily at the penumbra edge. The levels of M1 polarized microglia increase significantly 24 h after stroke onset and continue to rise.

The initial hours after cerebral ischemia typically show a dominance of M2-type microglia in terms of activation, which gradually shifts to M1-type over time (Hu et al., 2012; Kanazawa et al., 2017; Liu et al., 2018). In the 12 h following experimental middle cerebral artery occlusion (MCAO), a mix of M1 and M2 microglia can be observed (Zhu et al., 2019). After stroke, M1-type microglia are mainly concentrated in the penumbra within 24 h, and the level of its marker CD68 significantly increases from 24 h after MCAO. At a later time point, M1-type microglia can be observed in both the penumbra and the ischemic center (Ma et al., 2017). On the other hand, the expression of M2-type microglia in the acute stage of stroke is limited to the ischemic core area and reaches its peak 3–5 days after MCAO. It gradually decreases over time and changes into M1-type microglia in the infarct margin area. By day 14, the level of M2-type microglia has returned to the its pre-injury level (Mabuchi et al., 2000; Hu et al., 2012). However, it has also been reported that the expression of genes associated with M2 phenotype returns to baseline at 28 days after ischemia (Liu et al., 2018; Venkat et al., 2019). In contrast, M1-type microglia continue to increase for 14 days after MCAO (Perego et al., 2011; Hu et al., 2012). Figure 2 illustrates the dynamics of microglia polarization after cerebral ischemia. It is worth noting that the activation of microglia may differ between the permanent ischemia model and the transient cerebral ischemia reperfusion model. In the permanent occlusion model, activated microglia are primarily located in the penumbra rather in the infarct center. However, in transient ischemic animals, activated microglia are found in both the infarct center and the penumbra (Zanier et al., 2015).

In the late stage of MCAO, M1-type microglia-mediated inflammation can cause further damage to neurons and blood-brain barrier, exacerbating tissue destruction and functional deterioration (Wimmer et al., 2018). Recombinant human fibroblast growth factor 21 (rhFGF21) has been found to decrease M1-type polarization of microglia after cerebral ischemia, promote M1-to-M2-type polarization transformation, and activate PPARγ in the ischemic penumbra (Jiang et al., 2018; Wang D. et al., 2020). Hypothermia is also known to protect brain from ischemic stroke. In the MCAO model, maintaining a low temperature between 32 and 34°C can reduce the number of CD16-positive M1 microglia and increase the number of CD206-positive M2 microglia, thereby alleviating brain injury (Liu et al., 2018). This effect is likely related to the promotion of M1-to-M2-type polarization transformation of brain microglia in ischemic mice by low temperature.

After 24 h of cerebral ischemia in mice, CD11b-positive cells often gather around neurons in the ischemic core and peripheral regions, indicating microglia activation. CD11b/CD68 double-positive cells appear in both regions, with round cell bodies, while the CD68-positive branch protrusions wrap NeuN-positive cells, suggesting a potential role in engulfing neurons. In contrast, Ym1-positive cells are only found in the ischemic core, and CD11b/Ym1 double-positive cells do not seem to participate in neuronal phagocytosis (Perego et al., 2013). There are sex differences in CD11b expression in microglia in adult mice, with higher levels observed in female mice compared to males. However, only male mice show an increase in CD11b immunoreactivity after MCAO. This suggests that the sex difference of CD11b expression levels may influence the different phagocytosis responses of male and female microglia to ischemic injury (Morrison and Filosa, 2016).

The inflammatory environment undergoes changes over time and plays a crucial role in orchestrating microglial polarization. Inhibition of M1-type microglia polarization has been found to reduce neurological deficits following cerebral ischemia (Zhang et al., 2019; Mota et al., 2020), while promoting the polarization of M2-type microglia can aid in tissue remodeling and repair (Choi et al., 2017; Jiang et al., 2021). Future research should aim to regulate the balance between M2 and M1 phenotypes, maintain inflammatory defense, and promote neuroprotection, rather than simply reducing the damage caused by M1-related chronic inflammation (Lian et al., 2020).

## 5. Transformation of M1 and M2 phenotypes

Microglia activation serves as both the initiating and continuing factor of the inflammatory response in ischemic stroke. The M1/M2 phenotype represents two distinct activation states, but *in vivo*, microglia may exist in a spectrum of different and overlapping polarized phenotypes that can be reversed or transformed based on microenvironmental signals (Mosser and Edwards, 2008; Colton and Wilcock, 2010; Tanaka et al., 2020). Transcription regulators, cytokines, and inflammatory signaling pathways are involved in regulating the M1 and M2 phenotypic transformations. Therefore, future research should explore the complexity of microglia activation and its regulation, as well as identify potential therapeutic targets to modulate microglia activation states for optimal recovery after ischemic stroke.

### 5.1. Peroxisome proliferation-activated receptor γ (PPARγ)

Peroxisome proliferation -activated receptor γ is a ligand-dependent nuclear transcription factor that exists in multiple subtypes, including PPARγ. PPARγ is expressed in both neurons and microglia, where it exerts neuroprotective and anti-inflammatory effects on ischemic injury. It plays a key role in polarizing microglia from M1 type to M2 phenotypes (Yu et al., 2015; Li L. et al., 2021) and neutrophils from N1 to N2 phenotypes (Cuartero et al., 2013). PPARγ activation has been detected during M2 polarization of microglia (Zhou et al., 2020). In non-ischemic tissues, the expression of PPARγ is low, but it increases significantly in ischemic neurons, peaking at 24 h after MCAO (Victor et al., 2006). Troglitazone and Pioglitazone, which are classic insulin sensitizers, have been shown to reduce neuroinflammation and infarct volume in transient cerebral ischemia by targeting PPARγ activation (Sundararajan et al., 2005). Rosiglitazone, another PPARγ agonist, has been found to reduce neuroinflammation and autophagy-induced cell death in rats with global cerebral ischemia (Shao and Liu, 2015). RhFGF21, on the other hand, reduced neuroinflammation after cerebral ischemia by inhibiting NF-κB and upregulating PPARγ, thereby inhibiting M1 polarization and pro-inflammatory cytokine expression in microglia (Wang D. et al., 2020). However, some studies have shown that antagonistic or knockout of PPARγ can inhibit the activation of NF-κB-IKKβ by activating the LKB1-AMPK signal and promote the LPS-induced M1 to M2 phenotype transformation of microglia (Ji et al., 2018). Therefore, PPARγ may act as a hub molecule and play different roles in regulating polarization through different signaling pathways.

### 5.2. Interferon regulatory factor (IRF)

Interferon regulatory factor is a multifunctional transcription factor involved in interferon transcription regulation, signal transduction, and immune regulation. IRF5 and IRF4 mediate M1 and M2 polarization of microglia, respectively (Al Mamun et al., 2018). IRF5 and IRF4 balance each other, and reducing the expression of IRF5 can promote M2-type activation, increase IRF4 expression, inhibit the inflammatory response, and improve outcomes in cerebral ischemia. Conversely, reducing IRF4 expression can lead to increased IRF5 expression, promote M1-type activation, exacerbate the inflammatory response, and lead to worse outcomes in cerebral ischemia (Al Mamun et al., 2020). Age has an impact on the regulatory effect of IRF4/5 on stroke-related inflammation. In young mice, the levels of IRF4 and CD206, anti-inflammatory factors such as TGF-β, IL-4, and IL-10, are significantly higher in the brain compared to old mice after cerebral ischemia. Conversely, in old mice, the levels of IRF5 and MHCII in the brain, as well as pro-inflammatory factors such as TNF-α, iNOS and IL-6 in the serum, are significantly higher than in young mice (Zhao et al., 2017). The expression of IRF4/5 in microglia is significantly higher in aged female mice compared to male mice, and this difference is further amplified by cerebral ischemia injury. In ischemic brain tissue of elderly female mice, the IRF4 gene is inhibited while IRF5 gene is activated. Consequently, older females tend to have higher mortality and poorer outcomes after stroke. This sex-specific phenotype is associated with epigenetic modification of IRF4 and IRF5 by the escape genes kdm5c and kdm6a, respectively, on the X chromosome (Qi et al., 2021). When considering the regulation of microglia polarization by targeting IRF, appropriate intervention timing, gender, and age should be taken into account.

### 5.3. Toll-like receptor 4 (TLR4)

Toll-like receptor 4 is primarily expressed on microglia and plays a crucial role in recognizing damage-associated molecular patterns (DAMPs). It is also one of the primary receptors for LPS binding and can activate NF-κB pathway to regulate inflammatory processes. Polyphenolic natural products, including quercetin (Le et al., 2020), curcumin (Liu et al., 2017), resveratrol (Mota et al., 2020), baicalin (Yang et al., 2019), can regulate microglia polarization by inhibiting TLR4/NF-κB pathway, leading to anti-inflammatory roles in ischemic stroke (Li et al., 2022). Stimulation of the vagus nerve during the acute stage of cerebral ischemia can inhibit M1-type polarization and promote M2-type polarization by blocking the TLR4/MyD88/NF-κB signaling pathway, leading to improved outcomes in ischemic stroke (Zhang et al., 2021). Analgecine (AGC), an analgesic for lumbar pain in patients with spinal degenerative diseases, has been shown to inhibit M1-type polarization and promote M2-type polarization by inhibiting TLR4/MyD88/NF-κB signaling pathway, which reduced neuroinflammation during cerebral ischemia (Yang et al., 2021). TLR4 deficiency has been shown to regulate the polarization of microglia toward the M2 phenotype, improving neurological function following cerebral ischemia and traumatic brain injury (Yao et al., 2017; Tian et al., 2019). Therefore, TLR4 is a promising target for stroke therapy. ApTOLL is an aptamer that antagonizes TLR4. In Hernández-Jiménez et al. (2022), a dose-ascending, randomized, placebo-controlled Phase I clinical trial was conducted to evaluate the safety and pharmacokinetic profile of ApTOLL in healthy volunteers. In Hernández-Jiménez et al. (2023), a multicenter, double-blind, randomized, placebo-controlled Phase Ib/IIa clinical study (NCT04734548) was initiated to evaluate the tolerability, safety, pharmacokinetic, and biological effects of ApTOLL in patients with acute ischemic stroke (AIS) who are eligible for endovascular therapy (EVT). The ongoing clinical study will provide a basis for the clinical application of ApTOLL.

### 5.4. Galectins

Galectins are a family of carbohydrate-binding proteins that have been identified as potential regulators of microglia polarization, immune surveillance, and neuroinflammation (da Rosa et al., 2021). The Galectin family consists of 15 members, of which Gal-1,3,4,8,9 are expressed in the brain and involved in neural regulation. Deletion of Gal-1 significantly enhances microglia activation, while Gal-1 promotes microglia inactivation through glycosylation-dependent mechanisms, thus playing a neuroprotective role (Aalinkeel and Mahajan, 2016). Additionally, Gal-1 also targets activated microglia, promoting phagocytosis and transformation to the M2 phenotype (Rinaldi et al., 2016).

In a model of cerebral ischemia, Gal-3 expression is dependent on microglia activation (Rabinovich and Toscano, 2009). Gal-3 is an endogenous mediator of injury-induced microglia activation and proliferation (Ladeby et al., 2005; Lalancette-Hébert et al., 2007). Deletion of Gal-3 gene inhibits microglia activation (Espinosa-Oliva et al., 2021), resulting in insufficient microglia proliferation after ischemic injury, which leads to an increased size of infarction and number of apoptotic neurons (Lalancette-Hébert et al., 2012). In one study, administrating a single dose of Gal-3 recombinant protein into the lateral ventricle 24 h after stroke resulted in increased immune reactivity of Ym1 and up-regulated expression of anti-inflammatory factor IL-4, while reducing the immune reactivity of iNOS and the expression of pro-inflammatory factors (Rahimian et al., 2019). This suggests that Gal-3 can promote M2-type polarization of microglia after cerebral ischemia. However, other studies have found that Gal-3 released by microglia after global ischemic injury can act as an endogenous paracrine TLR4 ligand, activating TLR4 and resulting in sustained activation of microglia and increased neuronal damage (Burguillos et al., 2015). Additionally, a protective mechanism against cerebral ischemia has been found in Gal-3 knockout mouse model, particularly in the hippocampus and striate cortex (Doverhag et al., 2010). Therefore, the role of Gal-3 in neuroinflammatory response is complex, and it may play an crucial role in the fine-tuning of neuroinflammation (Rabinovich and Toscano, 2009).

### 5.5. Cysteinyl leukotriene receptor (CysLTR)

Cysteinyl leukotrienes (CysLTs) are potent lipid inflammatory mediators that activate their receptors (CysLTR1 and CysLTR2), mediating pro-inflammatory effects (Gelosa et al., 2017). During the pathological process, CysLTR1 and CysLTR2 expression levels in cerebral vascular endothelial cells, astrocytes, microglia, and neurons were significantly up-regulated. Upregulation of CysLTRs in activated microglia in the ischemic core of MCAO rats was associated with inflammation and exacerbation in the subacute phase (Shi et al., 2012; Mansour et al., 2018; Wang Y. et al., 2020). Inhibition of CysLTR1 or CysLTR2 protects against LPS or ischemia-induced microglial inflammation and brain injury (Shi et al., 2015; Chen F. et al., 2017; Lin et al., 2017). Neuroprotection is provided by the CysLTR1 antagonist Montelukast and the CysLTR2 antagonist HAMI3379, as they inhibit ischemia-induced microglial activation and pro-inflammatory cytokine release (Shi et al., 2015). The effect of CysLTR1 siRNA is limited in comparison to that of Montelukast, suggesting that Montelukast may produce neuroprotective effects through both CysLTr2-dependent and independent mechanisms (Theron et al., 2014; Marschallinger et al., 2015; Chen F. et al., 2017). Activation of CysLTR2 promotes M1-type polarization and inhibits M2-type polarization through NF-κB pathway (Wang Y. et al., 2020). Specific blocking of CysLTR2 can inhibit M1-type polarization and reduce ischemic neuronal damage (Zhang et al., 2013; Shi et al., 2015). IL-4, in combination with HAMI3379 (a CysLTR2 inhibitor), has been shown to promote M2-type polarization of microglia (Zhao et al., 2019). Additionally, mesenchymal stem cell-derived exosomes have been found to inhibit M1-type polarization of microglia by inhibiting CysLTR2, thereby alleviating cerebral ischemia reperfusion injury (Zhao et al., 2020a,b). The combination of baicalin and Geniposide (BC/GP) has been shown to reverse the polarization state of microglia by down-regulating 5-LOX/CysLTRs pathway, promoting the transformation of M1-type to M2-type polarization, and inhibiting cerebral ischemia-induced inflammation and injury (Wu et al., 2019). These results suggest that CysLTR1 and CysLTR2 play a role in microglia activation and neuroinflammation after cerebral ischemia and that CysLTR1 and CysLTR2 antagonists may serve as new anti-inflammatory agents for the treatment of microglia inflammation and neurotoxicity following cerebral ischemia.

### 5.6. Nuclear factor erythroid 2-related factor 2 (NRF2)

Nuclear factor erythroid 2-related factor 2 is a key regulatory factors of the endogenous antioxidant stress defense system. While no significant NRF2 immunopositive signal is observed in the infarct core area in the early stage after MCAO, NRF2 can be detected in microglia, astrocytes, and neurons in the penumbra and reaches peak levels 24 h after MCAO (Dang et al., 2012; Takagi et al., 2014). Activation of NRF2 has been shown to promote M2-type polarization of microglia after cerebral ischemia (Wang Y. et al., 2018; Hsia et al., 2020; He et al., 2021). Sevoflurane preconditioning can protect against cerebral ischemia by increasing the anti-inflammatory phenotype of microglia through activating the GSK-3β/NRF2 pathway (Cai et al., 2021). Similarly, Magnolol, and Dexmedetomidine have been found to promote M2-type polarization of microglia by activating the NRF2/HO-1/NLRP3 pathway (Tao et al., 2021; Wang et al., 2022). Non-mitogenic fibroblast growth factor 1 (nmFGF1) regulates microglia polarization by activating the NRF2 pathway and inhibiting the NF-κB pathway to protect against ischemic stroke (Dordoe et al., 2022). Tanshinol borneol ester (DBZ) promotes the transformation of microglia from the M1 to M2 phenotype by activating the Akt/GSK-3β/NRF2 pathway and releasing a series of anti-inflammatory and antioxidant factors to protect against cerebral ischemia (Liao et al., 2020). Sinomenine also promotes M2-type polarization by activating NRF2 and alleviates cerebral ischemia injury in rats (Bi et al., 2021). The ability of NRF2 to promote M2-type polarization in microglia, in addition to its antioxidant effects, makes it a promising target for treating ischemic stroke and other CNS diseases.

### 5.7. Interleukin 4 (IL-4)

Interleukin 4 is a unique cytokine that can regulate microglia function and promote brain repair (Zhao et al., 2015). IL-4 induces microglia M2 polarization and improves long-term neurological function after stroke (Liu et al., 2016). After cerebral ischemia, neurons in the penumbral zone rapidly produce IL-4. This IL-4 induces the activation of M2-type microglia, up-regulates the expression of IL-4 receptors and trophic factors on the surface of microglia, and activates PPARγ to enhance PPARγ-mediated phagocytosis of apoptotic neurons (Zhao et al., 2015). It is worth noting that neurons with excitotoxic damage due to overactivation of NMDA receptors also produce IL-4, promoting microglia to neuroprotective M2-type polarization (Ting et al., 2020). In female mice, IL-4 is required for neuroprotective effects, and female mice protected by IL-4 after focal cerebral ischemia show dominant M2 activation and reduced inflammatory infiltration (Xiong et al., 2015).

However, a study showed that the simultaneous loss of IL-4, IL-5, IL-9, and IL-13 did not affect neuropathologic responses in subacute and chronic ischemic stroke (Perego et al., 2019a). Perego et al. (2019a) investigated the phenotype changes of microglia at 24 h, 7 days, and 5 weeks after pMCAO using transgenic mice with simultaneous deletion of IL-4, IL-5, IL-9 and IL-13 genes (4KO). They found that CD16/32 (M1 marker) expression was lower but CD206 (M2 marker) expression was higher in 4KO mice than in wild-type mice at 24 h and 7 days after pMCAO. However, the neuropathological results of the 4KO mice did not differ from those of wild-type mice. This conclusion caused some controversy. Xu et al. (2019) suggested that IL-4 is induced by injured neurons in the ischemic penumbra region, and the use of pMCAO models with less penumbra tissue may result in negative results for differences between genotypes (Zhao et al., 2015). Perego et al. (2019b) argued that they demonstrated that Th2 cytokines, such as IL-4, are not necessary neural immune signals to drive the outcome of cerebral ischemia, although penumbra extension may be different in pMCAO and tMCAO models, and the results may differ in cerebral ischemia models with a higher degree of penumbra.

### 5.8. Signal transducer and activator of transcription (STAT)

Excessive production of pro-inflammatory cytokines, including IL-1, IL-6, and TNF-α, during the acute phase following focal cerebral ischemia, is a major contributor to inflammatory injury. The effects of these cytokines are magnified by the JAK-STAT signaling pathway downstream. Here, phosphorylated STAT is translocated to the nucleus and regulates gene expression downstream. STAT1, STAT3, and STAT6 are all involved in the activation of microglia induced by ischemia. Cerebral ischemia can trigger the phosphorylation of both STAT1 and STAT3. Phosphorylated STAT1 can exacerbate ischemia-induced neuronal injury, while STAT1 knockout can decrease cerebral infarction volume in mice (Takagi et al., 2002). STAT activation can induce the transformation of microglia into an M1 phenotype, while STAT6 activation can induce an M2 phenotype (Deszo et al., 2004). Minocycline, an antibiotic medication, can inhibit the phosphorylation of STAT1 and promote the phosphorylation of STAT6 after cerebral ischemia. As a result, it can regulate the M1/M2 polarization of microglia by modulating the STAT1/6 pathway (Lu et al., 2021).

During cerebral ischemia, STAT3 is expressed in activated microglia, astrocytes, and endothelial cells, which are closely associated with inflammation. By upregulating the phosphorylation level of STAT3 and increasing the levels of neurotrophic factors and anti-inflammatory cytokines, IL-10 can promote the polarization of M2c microglia and facilitate tissue repair (Miki et al., 2021). Fingolimod (FTY720) is a high-affinity agonist of sphingosine 1 phosphate (S1P) receptor and is the first FDA-approved drug for the treatment of relapsing-remitting MS due to its immunosuppressive action (Fu et al., 2015). By activating STAT3 signaling pathway, Fingolimod promotes M2-type microglia activation and oligodendrocyte regeneration, which helps improve white matter integrity and alleviate cognitive dysfunction caused by hypoperfusion (Qin et al., 2017).

It’s worth noting that the JAK2/STAT3 signaling pathway likely has a biphasic in regulating cerebral ischemia (Zhong et al., 2021). The overactivation of STAT3 induced by ischemia primarily occurs in microglia (Chen S. et al., 2017). Inhibition of JAK2 prevents the phosphorylation and nuclear translocation of STAT3, thereby reducing cerebellar infarct volume and improving neurological deficits (Satriotomo et al., 2006). Stachydrine inhibits the phosphorylation of JAK2/STAT3 and improves neural deficits in rats with MCAO (Li et al., 2020). On the other hand, Xuesaitong down-regulates the transcription level of STAT3, promoting M2-type polarization of microglia and providing neuroprotection in MCAO mice (Li et al., 2019). Additionally, long non-coding RNA nuclear enriched transcript 1 (NEAT1) plays a vital role in immune regulation. Cerebral ischemia can significantly increase NEAT1 expression levels. Knocking down NEAT1 inhibits the Akt/STAT3 signaling pathway, promotes M2-type polarization of microglia, inhibits M1-type polarization, and ultimately reduces neuronal apoptosis caused by ischemia (Ni et al., 2020; Lian and Luo, 2021).

### 5.9. Triggering receptor expressed on myeloid cells-2 (TREM2)

Triggering receptor expressed on myeloid cells-2 is a single transmembrane receptor primarily localized in microglia. Like other phagocyte receptors, TREM2 moves between the cytoplasm and membrane and regulates microglial phagocytosis, migration, and survival. Additionally, TREM2 can directly induce microglial phenotypic transformation. A lack of TREM2 is harmful to microglial phagocytosis of synaptic components, while overexpression of TREM2 can reduce the pro-inflammatory response of microglia (Zhang et al., 2017; Ren et al., 2018; Gratuze et al., 2020; Ruganzu et al., 2021).

Triggering receptor expressed on myeloid cells-2 may have a neuroprotective role in experimental ischemic stroke by reducing neuroinflammation (Kawabori et al., 2013; Wu et al., 2017). Its expression is highly increased in microglia following cerebral ischemia, and its level of expression can be used to predict the prognosis of cerebral ischemia, rather than its expression level in circulating macrophages (Kurisu et al., 2019). This supports the idea that phagocytosis is mainly mediated by microglia in the brain, rather than macrophages of bone marrow origin in tMCAO model (Schilling et al., 2005). Microglia with TREM2 knocked out show reduced activity after ischemia, and their phagocytosis of injured neurons is also reduced (Kawabori et al., 2015).

Triggering receptor expressed on myeloid cells-2 plays a role in regulating the M1 to M2 phenotype transformation of microglia after ischemic stroke. In mouse models of MCAO, TREM2 activation and up-regulation promote the transition of microglia from a pro-inflammatory M1 phenotype to an anti-inflammatory M2 phenotype. Administering systemic TREM2 agonists or injecting TREM2 lentivirus directly into the ventricle accelerates neurological recovery in mice. These findings suggest that TREM2 may modulate microglial phenotypes after stroke, affecting the short-term prognosis of stroke in mice (Zhai et al., 2017).

Triggering receptor expressed on myeloid cells-2 has the ability to bind to different ligands, which regulates the direction of TREM2 signal transduction and produces different effects. As a novel regulator of microglial phenotype, TREM2 represents an attractive target for microglial regulation in the treatment of ischemic stroke. However, the mechanism and temporal dynamics of TREM2 in regulating phenotypes after stroke are currently unclear and require further investigation.

### 5.10. Cathepsin

Cathepsins are a group of cysteine proteolytic enzymes in the endosomal lysosome system that play a crucial role in the lysosomal protein degradation pathway. Activated microglia have a complex role that is related to its phenotypic changes, and the expression and secretion of various cathepsins are important in the M1-type polarization of microglia, chronic neuroinflammation, and neuronal death. The cathepsins associated with microglia polarization and neuroinflammatory regulation include Cathepsin B, C, E, and H (Nakanishi, 2020).

Cathepsin B in microglia can activate NLRP3 inflammasome, leading to the activation of caspase-1 and the subsequent release of IL-1β. Alternatively, it can also directly activate procaspase-11, independent of NLRP3, leading to the activation of caspase-1 and release of IL-1β from microglia (Sun et al., 2012; Chen et al., 2018; Campden and Zhang, 2019). After hypoxic ischemia, the deficiency of Cathepsin B results in a transient polarization of microglia toward the neuroprotective M2 phenotype instead of the M1 type (Ni et al., 2015). NF-κB is a crucial transcription factor that regulates multiple genes involved in M1-type polarization in microglia. Its activity is higher in M1-polarized microglia but lower in M2-polarized microglia (Karin, 2006; Biswas and Lewis, 2010; Dorrington and Fraser, 2019). NF-κB inhibitor α (IκBα) is an endogenous negative regulator of NF-κB, which prevents its translocation into the nucleus. IκBα undergoes proteolytic degradation upon signal induction, which is mediated by the ubiquitin-proteasome system. This degradation leads to the rapid and transient activation of NF-κB. During the early stage of hypoxic-ischemic brain injury, ubiquitinated IκBα is proteolytically degraded by the proteasome, which results in the activation of NF-κB (He et al., 2019). After the early activation of NF-κB, Cathepsin B in microglia autophagolysosomes induces delayed but persistent activation of NF-κB by degradation of IκBα. Subsequently, NF-κB further enhances the expression of Cathepsin B and Beclin 1, a key regulator of autophagy, which promotes autophagy in post-ischemic brain injury (Ni et al., 2015). Cathepsin B has been shown to play a role in the phenotypic switch of microglia from M2 to M1 by modulating the activity of NF-κB.

Cathepsin C is primarily induced in activated microglia after systemic injection of LPS, promoting microglia polarization toward neurotoxicity and exacerbating neuroinflammation. This effect is mainly achieved through the activation of the Ca^2+^ dependent protein kinase C/p38 MAPK/NF-κB pathway (Liu et al., 2019). The interaction between Cathepsin C and inflammatory cytokines in microglia amplifies neuroinflammation-related cellular events and is considered one of the underlying causes of persistent inflammation in the brain.

Cathepsin E is not normally detected in healthy brains and is only expressed in activated microglia in pathological brains, suggesting that its enzymatic activity is associated with activated microglia (Nakanishi et al., 1993; Ni et al., 2015). In neonatal mice with hypoxic-ischemic brain injury, activated microglia secreted Cathepsin E to hydrolyze tumor necrosis factor-associated apoptosis inducing ligand (TRAIL) on the surface of microglia. This leads to the activation of NF-κB via TRAIL death receptor DR5, which promotes M1-type polarization of microglia (Ni et al., 2015).

Interferon-β (IFN-β) secreted by microglia is known to have a neuroprotective effect (Lobo-Silva et al., 2017; Scheu et al., 2019). Deficiency of Cathepsin H leads to decreased levels of TLR3 and IFN-β expressed in splenic dendritic cells (Okada et al., 2018). TLR3 is involved in the activation of NF-κB and IRF3, which leads to the production of IFN-β and pro-inflammatory cytokines. The proteolysis of TLR3 is essential for its function, and Cathepsin H and B play a crucial role in the regulation of this process (Garcia-Cattaneo et al., 2012). In neonatal mice, the deficiency of Cathepsin H leads to prolonged neurotoxic M1-like polarization following hypoxia ischemia, which ultimately results in microglia death and subsequent neurotoxic A1 astrocyte proliferation and progressive neuronal death (Liddelow et al., 2017; Ni et al., 2021). However, in mice injected with LPS, upregulated expression of Cathepsin H in microglia exacerbates neuronal death in neuroinflammation (Fan et al., 2015). Based on the current evidence, it appears that maintaining appropriate levels of Cathepsin H may be important in regulating neuroinflammation and protecting against neurological damage. Both deficient and elevated levels of Cathepsin H have been associated with adverse effects on neuroinflammation, suggesting that a balanced level of Cathepsin H activity is necessary for maintaining healthy brain function. Further research is needed to fully understand the complex role of Cathepsin H in neuroinflammation and to explore potential therapeutic strategies targeting this enzyme in neurological disorders.

**FIGURE 3:**
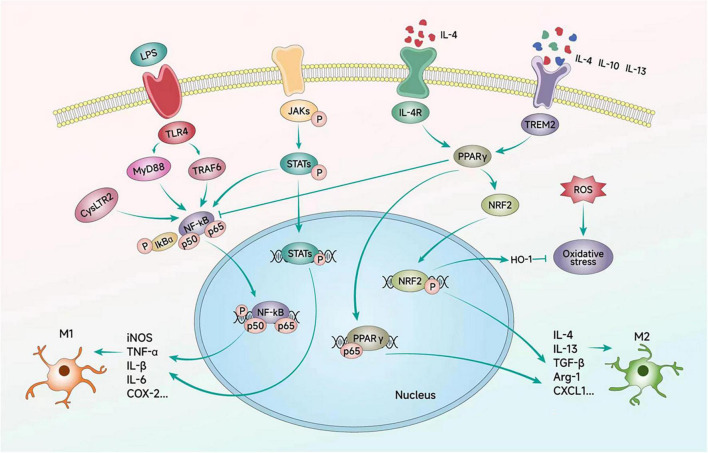
Signaling pathways regulating microglial polarization. Microglial polarization is triggered by extracellular factors such as LPS, cytokines, and ILs, which bind to their corresponding receptors on the microglial cell membrane. These signaling events initiate downstream cascades that transfer signals to the nucleus and regulate the expression of inflammatory factors through a series of transcription factors. Ultimately, these signaling pathways contribute to the emergence of different polarized phenotypes of microglial cells.

Figure 3 and Table 2 summarizes the molecules that regulate microglia polarization and their functions. These include various transcription factors, cytokines, chemokines, and receptors, which can promote either M1 or M2 polarization. Table 3 summarizes the factors and drugs that can modulate microglia polarization phenotypes. These include various natural compounds, such as curcumin and resveratrol, as well as drugs that are commonly used in clinical settings, such as minocycline and dexamethasone. These factors and drugs have been shown to promote either M1 or M2 polarization, and their effects on microglia polarization may have important implications for the treatment of neurological disorders.

**TABLE 2:** Regulatory molecules and their functions in microglia polarization.

Molecules	Localization	Functions	References
Cathepsin	Lysosome	Inhibition of Cat B: M2↑ Production of Cat C: M1↑ Expression of Cat E: M1↑ Deficiency of Cat H: microglial cell death↑ Up-regulation of Cat H: M1↑	Sun et al., 2012; Fan et al., 2015; Ni et al., 2015, 2021; Liddelow et al., 2017; Chen et al., 2018; Campden and Zhang, 2019; Liu et al., 2019
CysLTR	Nucleus/membrane	Inhibition of CysLTR: M1↓, M2↑	Shi et al., 2012; Zhang et al., 2013; Theron et al., 2014; Marschallinger et al., 2015; Shi et al., 2015; Chen F. et al., 2017; Lin et al., 2017; Mansour et al., 2018; Wu et al., 2019; Zhao et al., 2019, 2020a; Wang Y. et al., 2020
Galectin 3	Cytoplasm	M2↑ M1↑	Doverhag et al., 2010; Lalancette-Hébert et al., 2012; Burguillos et al., 2015; Rahimian et al., 2019
IL-4	Cytoplasm	M2↑	Xiong et al., 2015; Zhao et al., 2015; Ting et al., 2020
IRF	Nucleus/cytoplasm	IR5↓→IRF4↓→M2↑ IR4↓→IRF5↓→M1↑	Al Mamun et al., 2020
Nrf2	Nucleus/cytoplasm	Activation of Nrf2: M2↑	Takagi et al., 2014; Wang Y. et al., 2018; Hsia et al., 2020; Liao et al., 2020; Bi et al., 2021; Cai et al., 2021; He et al., 2021; Tao et al., 2021; Dordoe et al., 2022; Wang et al., 2022
PPARγ	Nucleus/cytoplasm	Activation of PPARγ: M2↑ Inhibition of PPARγ: M2↓	Sundararajan et al., 2005; Victor et al., 2006; Cuartero et al., 2013; Shao and Liu, 2015; Yu et al., 2015; Ji et al., 2018; Wang D. et al., 2020; Zhou et al., 2020; Li L. et al., 2021
STAT	Nucleus/cytoplasm	Activation of STAT1: M1↑ Activation of STAT6: M2↑ Activation of STAT3: M2c↑, M2↑ Inhibition of STAT3: M1↓	Takagi et al., 2002; Deszo et al., 2004; Satriotomo et al., 2006; Chen S. et al., 2017; Qin et al., 2017; Li et al., 2019; Li et al., 2020; Ni et al., 2020; Lian and Luo, 2021; Lu et al., 2021; Miki et al., 2021
TLR4	Cell membrane	Inhibition of TLR4: M2↑, M1↓	Liu et al., 2017; Yao et al., 2017; Tian et al., 2019; Yang et al., 2019; Mota et al., 2020; Le et al., 2020; Yang et al., 2021; Zhang et al., 2021; Li et al., 2022
TREM2	Cell membrane	Activation of TREM2: phagocytosis↑, M2↑	Schilling et al., 2005; Kawabori et al., 2015; Zhai et al., 2017; Kurisu et al., 2019

**TABLE 3:** Pharmacological modulators of microglial polarization.

Drugs	Experimental models	Ways of administration	Targets	Functions	References
Analgecine (AGC)	MCAO	i.v. daily starting 3 h after MCAO.	TLR4	Inhibiting TLR4/MyD88/NF-κB to inhibit M1-type polarization.	Yang et al., 2021
Baicalein	MCAO	i.g. after reperfusion and continued once a day for 3 days.	TLR4, STAT1	Suppressing TLR4/NF-κB pathway and down-regulate phosphorylated STAT1 to Promote the phenotypic shift of microglia from M to M2.	Ran et al., 2021
Curcumin	SAH	i.p. at 15 min post SAH induction.	TLR4	Inhibiting TLR4/MyD88/NF-κB to promote microglia phenotype shift toward M2.	Gao et al., 2019
Dexmedetomidine	pMCAO	i.v. at the beginning of the operation.	NRF2	Activating Nrf2/HO-1 to promote M2-polarization of microglia.	Zhang et al., 2016
Fingolimod	White matter ischemic injury	i.p. for 3, 10 or 30 consecutive days.	STAT3	Promoting the shift of microglia from M1 to M2 polarization via STAT3 signaling.	Qin et al., 2017
Ginsenoside Rd	MCAO	i.p. injection 4 h after MCAO.	NF-κB	Suppresses proinflammatory M1-polarization of microglia	Zhang et al., 2016
HAMI3379	LPS-induced microglia	Supplement in cell culture medium.	CysLTR2	Blocking CysLTR2 to inhibit M1 polarization and promote M2 polarization.	Zhao et al., 2019
Magnolol	unpredictable mild stress induced depression	i.g.	NRF2	Upregulating Nrf2/HO-1 to promote M2 polarization.	Tao et al., 2021
Minocycline	MCAO/R	i.p. daily for 2 weeks.	STAT1/6	Regulating M1/M2 microglia polarization	Lu et al., 2021
	retinal IR	i.p. twice daily.	IL-4	Inducing M2 polarization of microglia	Ahmed et al., 2017
	ICH	i.p. twice daily.	TrkB/BDNF	Inducing activated M1 microglia into M2 neurotrophic phenotype.	Miao et al., 2018
MTK	MCAO	i.p. starting form 3 days before MCAO until sacrifice.	CysLTR1	Inhibiting CysLTR1 to enhance M2 polarized microglia.	Gelosa et al., 2019
rGal-3	MCAO	i.c.v. at 24 h after MCAO.	TLR4	Fine-tuning the polarization of microglia to an anti-inflammatory phenotype.	Rahimian et al., 2019
Resveratrol	OGD/R	Supplement in cell culture medium.	NRF2	Regulating M1/M2-type polarization of microglia.	Liu et al., 2022
rhFGF21	MCAO	i.p. daily starting at 6 h after reperfusion.	PPARγ, NF-κB	Upregulating PPARγ and suppress NF-κB to inhibit M1 polarization.	Wang D. et al., 2020
Rosiglitazone	global cerebral ischemia	i.p.	PPARγ	Activating PPARγ to promote M2 polarization.	Shao and Liu, 2015
Salvianolic acids	MCAO/R	i.p.	TLR4, STAT3	Switching M1/M2 phenotypes and inhibiting NLRP3.	Ling et al., 2021; Ma et al., 2021
Sinomenine	MCAO	i.p. 6 h after MCAO surgery daily for 3 days.	NRF2, NF-κB	Promoting Nrf2 and inhibit NF-κB to regulate microglia M1/M2 polarization.	Bi et al., 2021
T0070907	LPS-induced microglia	Supplement in cell culture medium.	PPARγ	Antagonizing PPARγ to promote M1-to-M2 phenotypic shift and microglial autophagy.	Ji et al., 2018
DBZ	MCAO	i.p. 1 h before, 4 h after cerebral ischemia, and daily for another 7 days.	NRF2	Promoting nuclear accumulation and stabilization of Nrf2 via the Akt (Ser473)/GSK3β(Ser9)/Fyn pathway to regulate microglia polarization.	Liao et al., 2020
Xuesaitong	MCAO	i.v.	STAT3	Modulating microglial phenotype and suppressing inflammation	Li et al., 2019; Zhu T. et al., 2021

## 6. Autophagy and ischemia microglia polarization

The transition of cells from one phenotypic state to another is closely linked to the rapid degradation of protein involved in the previous homeostatic state. Autophagy is a major mechanism of large-scale protein degradation in eukaryotic cells and a critical step in cell reprogramming (Zubova et al., 2022). Following cerebral ischemia, autophagy can be either activated or inhibited in various cell types in the brain, including neurons, astrocytes, vascular endothelial cells, and microglia. Autophagy plays a role in the regulating inflammatory responses, blood-brain barrier permeability, and microglia polarization (Lu et al., 2022). Indeed, in many CNS diseases, autophagy dysfunction and neuroinflammation are intertwined and mutually affect each other (Plaza-Zabala et al., 2017).

### 6.1. Autophagy participates in regulating microglial inflammation

Lipopolysaccharide can activate PI3K/Akt/mTOR pathway in microglia, which inhibits autophagic flow and enhances the inflammatory response (Ye et al., 2020). Conversely, autophagy activation can inhibit the increase of LPS-induced microglia pro-inflammatory factors, such as iNOS and IL-6 expression, and thus suppress the inflammatory response (Han et al., 2013). Annexin-A1 (ANXA1) mediates the degradation of IκB kinase-α (Iκk-α) via selective autophagy, which is promoted by the interaction between ANXA1 and the autophagy receptor NBR1. This inhibits the activation of the NF-κB signaling pathway and reduces the production of pro-inflammatory factors in OGD/R-damaged microglia (Li X. et al., 2021). Additionally, selective autophagy regulated by Beclin1 is involved in the degrading of the NLRP3 inflammasome, which regulate the production of IL-1β and IL-18 and affects the activation of microglia. Therefore, impaired autophagy in microglia can contribute to the neuroinflammation (Houtman et al., 2019). Autophagy can act as a negative regulator of inflammation in acute cerebral ischemia by participating in the inhibition of NLRP3-mediated neuroinflammation through the involvement of Beclin1, LC3, and p62 (Fu et al., 2020; Huang et al., 2021; Lugovaya et al., 2022).

### 6.2. Autophagy affects the function of microglia

Beclin 1 plays a crucial role in promoting effective microglial phagocytosis *in vitro* and in the mouse brain. The decreased levels of autophagy in microglia in AD are associated with reduced ability to clear abnormal protein accumulation, which highlights the importance of autophagy in maintaining protein homeostasis (Lucin et al., 2013). Deletion of the microglia-specific autophagy regulator Atg7, instead of the typical macrophage protein ULK1, may cause progressive MS-like disease (Berglund et al., 2020). Moreover, microglial autophagy also regulates neuroinflammation and tau protein pathology. Atg7 deletion in microglia leads to a pro-inflammatory transformation of microglia and enhances the pathology and diffusion of tau protein in neurons (Xu et al., 2021). Microglial Atg5 deficiency results in PD-like symptoms in mice, which could be attributed to the up-regulation of pro-inflammatory macrophage migration inhibitory factor (MIF) expression in microglia due to autophagy dysfunction (Cheng et al., 2020).

### 6.3. Autophagy regulates microglia polarization

Autophagy is a crucial homeostasis mechanism that regulates microglial activation states. It can modulate the phenotype of OGD-damaged microglia by regulating the balance between NF-κB and cAMP-response element binding protein (CREB) (Xia et al., 2016). Molecular switches, including TLR4, STATs, and PPARγ, play a crucial role in regulating autophagy-induced phenotypic changes. After ischemic injury, TLR4 expression in microglia is significantly increased, resulting in the accumulation of autophagosomes in microglia. However, knocking out of TLR4 or using autophagy inhibitors can induce the transformation of microglia from pro-inflammatory M1 phenotype to anti-inflammatory M2 phenotype, thereby exerting a neuroprotective effect (Qin et al., 2018). Nevertheless, heavily activated autophagy can clear the anti-inflammatory phenotype of TLR4-deficient microglia (Qin et al., 2018). These findings suggest that the maintenance of appropriate autophagic flux is critical for regulating microglial functional phenotypes. LPS-induced upregulation of STAT1 expression and downregulation of STAT6 expression in microglia, which can be reversed by autophagy inhibitors (Qin et al., 2018). The MS drug Fengomode inhibits LPS-induced microglial autophagy and STAT1 activation and promotes the transformation of M1-type microglia into M2 type microglia (Hu et al., 2021). Specific inhibition of PPARγ can stimulate the formation of microglial autophagosomes and their degradation by lysosomes, promoting the transformation of microglia from M1 to M2 type (Ji et al., 2018).

The polarization of microglia affects autophagic flux as well. In the context of cerebral ischemia, activated M1-type microglia expresses the phosphodiesterase enzyme 1 (PDE1), and inhibiting PDE1 enhances autophagy in microglia. The PDE1 inhibitor Vinpocetine inhibits M1-type polarization and promotes M2-type polarization in microglia in an autophagy-dependent manner. However, inhibiting microglia autophagy can counteract the regulatory effect of Vinpocetine on polarization (Zang et al., 2020).

Notably, the M2 polarization of microglia is highly dependent on autophagy (Sanjurjo et al., 2018). Treatment with IL-4 enhances the autophagic flux of microglia, leading to autophagy-dependent M2 polarization (Tang et al., 2019). Inhibition of autophagy by chloroquine significantly reduces the expression of M2-specific genes, such as Arg-1, FIZZ1, and IL-10, in wild-type macrophages, but this effect is not observed in Cathepsin S^–/–^ macrophages (Yang M. et al., 2014). This suggests that the regulation of microglia M2 polarization by autophagy may be linked to Cathepsin S.

### 6.4. Autophagy regulates microglia polarization at different phases after cerebral ischemia

The regulation of microglia autophagy flux and phenotype is dependent on the timing after ischemia and hypoxia. Studies have shown that the autophagy flux of microglia increases at 12, 24, and 48 h after OGD reperfusion but decreases at 72 h, during which most microglia show the M1 phenotype. If autophagy is inhibited by the lysosome protease inhibitor ammonium chloride (NH_4_Cl) 24 h after reperfusion of OGD, most microglia also show the M1 type (Xia et al., 2016). As mentioned earlier, the M2 phenotype is dominant within 24 h after ischemia reperfusion injury, indicating that autophagy flux can be appropriately increased at this stage to promote M2-type polarization of microglia. However, excessive autophagy can also lead to autophagic apoptosis or death of microglia. The M2 phenotype gradually decreases within 72 h after ischemia reperfusion, which may be related to the decrease of autophagy flux. Thus, it is crucial to maintain an appropriate level of autophagy flux at different phases after ischemia to regulate microglia polarization.

### 6.5. Key signaling molecules associated with autophagy and microglia polarization

Neuroinflammation and autophagy dysfunction are closely linked to the onset and progression of CNS diseases. However, the precise role of autophagy in microglia and neuroinflammation remains unclear. The regulation of microglial autophagy involves several critical factors, including mammalian target of rapamycin (mTOR), hypoxia-inducible factor-1 (HIF-1), and endoplasmic reticulum (ER) stress. These signaling pathways play a crucial role in modulating the autophagy flux and polarization of microglia, and dysfunction of these pathways has been implicated in the pathogenesis of various CNS disorders. Therefore, a better understanding of the molecular mechanisms underlying autophagy dysregulation in microglia could provide novel therapeutic targets for the treatment of neuroinflammatory diseases.

Mammalian target of rapamycin is a key regulator of cell metabolism and plays a critical role in modulating autophagy. mTOR exists in two signaling complexes, mTORC1 and mTORC2. While mTORC1 integrates various growth factor signals to promote protein, lipid, and nucleotide synthesis, it also blocks catabolic processes such as autophagy by post-transcriptional and transcriptional mechanisms (Laplante and Sabatini, 2013).

Activated mTORC1 is closely associated with autophagy induction. However, most autophagy-inducing conditions, such as nutrient or growth factor deprivation and low cellular energy levels, inhibit mTORC1 activity. mTORC1 inhibits autophagy induction by phosphorylation-dependent inhibition of unc-51-like kinase 1/2 (ULK1/2) and phosphatidylinositol 3-kinase catalytic subunit type 3 (Vps34). Additionally, it prevents overall expression of lysosome and autophagy genes by phosphorylating transcription factor EB (TFEB), which strictly regulates autophagy (McAlpine et al., 2013; Munson et al., 2015; Napolitano et al., 2018). The molecular mechanisms of upstream effector regulation of mTORC2 remain unclear. It is speculated that mTORC2 may indirectly inhibit autophagy by activating mTORC1 (Oh et al., 2010).

In transgenic mice with deletions of microglia-regulated proteins associated with mTOR, the inhibition of mTORC1 signaling significantly reduces the production of pro-inflammatory cytokines and chemokines, as well as the number of M1-type microglia, resulting in a significant reduction in infarct volume after MCAO (Li et al., 2016). TNF-α inhibits microglial autophagy through the Akt/mTOR signaling pathway, and enhancing autophagy promotes microglial polarization toward the M2 phenotype and inflammation regression (Jin et al., 2018). Sestrin2 plays a neuroprotective role in ischemic stroke mice by inhibiting mTOR and restoring autophagy flux to drive microglia toward the M2 phenotype (He et al., 2020).

Hypoxia-inducible factor-1 is a key transcription factor that regulates the response to anoxic environment. It is a heterodimer consisting of a functional α subunit and a structural β subunit, which is regulated by oxygen levels. During oxidative stress, HIF-1α forms a heterodimer with HIF-1β, translocates to the nucleus, binds to hypoxia response elements (HREs) in downstream gene promoters, and activates downstream BNIP3. BNIP3 can competitively bind with Beclin-1 to Bcl-2, releasing Beclin-1, a key autophagy protein, and inducing autophagy. Additionally, BNIP3 inhibits Rheb activity, which inhibits the mTOR pathway and promotes autophagy (Xu et al., 2020; Abo El-Ella, 2022). Hypoxia upregulates HIF-1α expression and increases autophagy flux in microglia, while the number of autophagosomes decreases significantly in cells transfected with HIF-1α-siRNA after hypoxia treatment. Pharmacological inhibition of HIF-1α can also down-regulate autophagy of hypoxic microglia (Wang et al., 2017). These findings suggest that HIF-1α mediates autophagy in microglia under hypoxia conditions, and it may be a regulatory target to maintain appropriate autophagy flux in microglia under such conditions.

**FIGURE 4:**
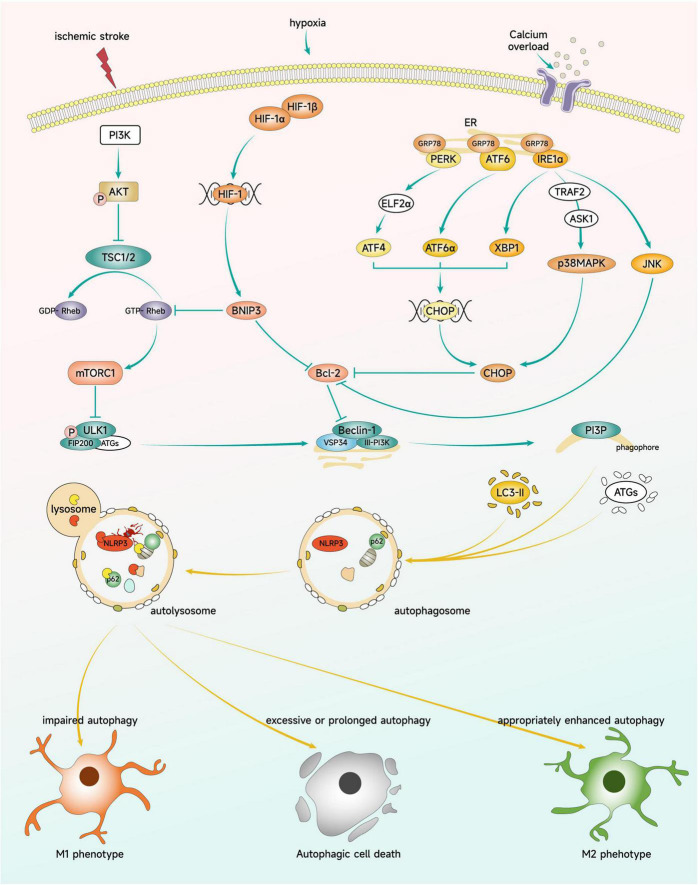
Key signaling pathways regulating microglial polarization through autophagy. Brain ischemia triggers signaling through mTOR, HIF-1, and ER stress pathways. These signaling molecules can impact microglial autophagy levels and modulate the polarization phenotype and cellular fate of microglia.

Ischemic stroke leads to Ca^2+^ overload, which causes an increase in protein misfolding and results in ER stress. When ER stress occurs, GRP78 releases PERK, IRE1, and ATF6,which activate the UPR and induce autophagy to degrade misfolded or unfolded proteins, thereby mitigating ER stress (Chaudhari et al., 2014). During this process, oligo PERK phosphorylates EIF2α and selectively translates ATF4 to induce autophagy gene expression. Oligo IRE1α binds TRAF2, activating ASK1 and its downstream kinase p38 MAPK and JNK. Furthermore, the intrinsic ribonuclease activity of IRE1α enables it to selectively cut and activate XBP1, a transcription factor that induces UPR gene expression. ATF6 is transported to the Golgi apparatus for proteolysis, releasing active AtF6α. ATF4, XBP1, and ATF6α converge on the promoter of CHOP gene, stimulating CHOP activation. CHOP can inhibit the expression of Bcl-2 gene, while JNK can directly inhibit the activity of Bcl-2, thus reducing the inhibition of Bcl-2 on the autophagy protein Beclin-1 and promoting autophagy (Han et al., 2022). Following OGD/R injury, autophagy and ER stress are significantly increased in microglia. SC-222227, a protein tyrosine phosphatase 1B inhibitor, can inhibit microglia autophagy by suppressing PERK-regulated ER stress, indicating the involvement of ER stress in the regulation of microglia autophagy during cerebral ischemia (Zhu Y. et al., 2021). Figure 4 illustrates the key signaling pathways by which microglia autophagy participates in the regulation of polarization.

The mechanism of microglial autophagy in ischemic stroke and its impact on stroke outcome have yet to be fully elucidated. Activated microglia autophagy may influence phenotype changes while serving a scavenging role (Chen et al., 2016), and may also contribute to nerve injury (Yang Z. et al., 2014; Qin et al., 2018). The dynamic shift of microglia polarization phenotype is a well-known factor in nerve repair following ischemic stroke. By introducing the appropriate subtype at the appropriate time, it may be possible to enhance CNS repair.

## 7. Conclusion and perspective

Despite significant progress, there are still important questions that need to be addressed in understanding the regulation of microglia polarization. For instance, how to effectively regulate the polarization of microglia using a large number of different types of receptors and signaling molecules, and how to adjust it over time during the progression of the disease to achieve the best balance between M1/M2 polarization. Furthermore, while modulation of microglia polarization shows promise for treating CNS diseases, its clinical application remains a significant challenge. The effects of many inhibitors or inducers of M1 or M2 polarization phenotypes are not simply all-or-nothing, but are dependent on time and dose. Moreover, it should be noted that M1-mediated inflammatory responses also have potential benefits that should not be simply suppressed or eliminated. It is also important to consider that maintaining a high level of M2-type polarization for a long time may have negative consequences, such as increased susceptibility to tumor growth, as M2-type macrophages have been shown to promote tumor growth.

Recent studies have been attempted to manipulate the anti-inflammatory polarization of microglia at the site of stroke, with promising results (Li Y. et al., 2021). Li Y. et al. (2021) designed ultrasound controlled, platelet-decorated microglia targeting therapy for ischemic stroke. Using a platelet-hybridized microglia platform, microglia can adhere strongly to injured cerebral vessels through platelet membrane infusion and achieve on-demand anti-inflammatory polarization by decorating with ultrasound-responsive IL-4 liposomes. This microglia platform demonstrates anti-inflammatory polarization at the stroke site after i.v. injection, promoting endogenous M2-type polarization of microglia and aiding in long-term stroke recovery. These results provide inspiration for the future applications of microglia polarization in the targeted regulation of CNS ischemia injury.

## Author contributions

HW and QC wrote the initial draft. JL, HZ, MW, LX, and YW contributed to reviewing the literature. QC designed the manuscript and prepared the final version. All authors read and approved the final manuscript.
